# Disclosing non-visible disabilities in educational workplaces: a scoping review

**DOI:** 10.1093/bmb/ldae004

**Published:** 2024-03-02

**Authors:** Juliet Hassard, Mehmet Yildrim, Louise Thomson, Holly Blake

**Affiliations:** Queen’s Business School, Queen’s University Belfast, Belfast, BT9 5EE, UK; School of Health Sciences, University of Nottingham, Nottingham, NG7 2RD, UK; School of Medicine, University of Nottingham, Nottingham, NG7 2DR, UK; School of Health Sciences, University of Nottingham, Nottingham, NG7 2RD, UK; NIHR Nottingham Biomedical Research Centre, Nottingham, NG7 2RD, UK

**Keywords:** disability disclosure, education, workplaces

## Abstract

**Introduction:**

a sizable proportion of the working population has a disability that is not visible. Many choose not to disclose this at work, particularly in educational workplaces where disability is underrepresented. A better understanding of the barriers and facilitators to disclosure is needed.

**Sources of data:**

this scoping review is based on studies published in scientific journals.

**Areas of agreement:**

the reasons underpinning disclosure are complex and emotive-in-nature. Both individual and socio-environmental factors influence this decision and process. Stigma and perceived discrimination are key barriers to disclosure and, conversely, personal agency a key enabler.

**Areas of controversy:**

there is a growing trend of non-visible disabilities within the workplace, largely because of the increasing prevalence of mental ill health. Understanding the barriers and facilitators to disability disclosure is key to the provision of appropriate workplace support.

**Growing points:**

our review shows that both individual and socio-environmental factors influence choice and experience of disclosure of non-visible disabilities in educational workplaces. Ongoing stigma and ableism in the workplace, in particular, strongly influence disabled employees’ decision to disclose (or not), to whom, how and when.

**Areas timely for developing research:**

developing workplace interventions that can support employees with non-visible disabilities and key stakeholders during and beyond reasonable adjustments is imperative.

## Introduction

People with disabilities are one of the world’s largest minority groups.^1^ Unfortunately, many continue to be overlooked, including in workplace settings.^2^ In the UK, one in five working-age adults report a disability, chronic health condition or neurodivergence.^3^ Over the last decade, an increasing proportion of working-age adults report having a long-term health condition or disability. This upward trend is understood to be driven by increasing rates of ‘non-visible’ disabilities (e.g. mental health conditions).^3^^,^^4^ Non-visible disabilities refer to physical, mental or neurological conditions that pose challenges to an individual’s movement, senses or activities, but may not be immediately or obviously observed.^4^ Examples include mental health conditions, autism, sensory processing difficulties, cognitive impairment (e.g. dementia and traumatic brain injury), ‘non-visible’ physical health conditions (e.g. chronic pain, diabetes), hearing loss and low or restricted vision. Various terms have been used to describe this broad category of disabilities (including, hidden, invisible and non-visible disability). In the context of this study, we use the term non-visible disability in line with UK government guidance.^5^

Disabled people, including those with non-visible disability, continue to face significant and diverse barriers to full participation in employment and inclusion at work.^4^^,^^6–8^ The disability employment gap (i.e. the difference in employment rates between disabled and non-disabled people) is pervasive and exists globally.^6^ In the UK, for example, 52.7% of disabled people were employed in 2021, compared with 81% of non-disabled people.^3^ There exists a ‘disability disclosure gap’ in the workplace, which is also sizable and, for many, a significant barrier to the promotion of their health, inclusion at work and quality of life.^9^ A 2017 survey conducted in the USA observed that 30% of employees reported a disability, chronic health condition or neurodivergence, but only 3.2% disclosed this to their employer.^10^ Research from the UK shows that around 40%^11^^,^^12^ of disabled workers felt uncomfortable discussing their disability at work, reporting concerns regarding career progression and anticipated stigma.^11^

Traditionally, much of the literature on employment and disability has not focused on the disclosure of non-visible disability to employers.^13^ Particularly when employees are seeking workplace accommodations and adaptations.^13^ However, growing evidence highlights the personal and emotive nature of disability disclosure in the workplace, and there is an increased understanding of the personal and system-level barriers and facilitators. This knowledge demonstrates the importance of employees’ personal experience and impact of this on the disclosure process. Existing reviews have explored disclosure considerations, although this has typically been focused on specific conditions (e.g. mental ill health^14^) rather than across the wider category of non-visible disabilities. This approach misses shared experiences across non-visible disabilities and health conditions. The current review will help to address this gap in knowledge.

The education sector has been selected, as it is characterized by an underrepresentation of disabled employees as compared with other sectors. In the UK, 23% of working age people reported a disability.^15^ In contrast, only 6.3% of academics and 8.5% of non-academics, in 2021/2022, declared having a disability.^16^ The School Workforce Census in 2023 found that disability data were not obtained for over half of teachers (53%), with reporting rates found to be substantially lower than other protected characteristics (e.g. gender and age).^17^ Similar trends have been found internationally for education (e.g. Canada^18^ and Australia^19^).

Therefore, we focus our review on educational workplaces to explore disabled employees’ experiences, within an industry characterized by challenges surrounding inclusion and representation. Empirically, this review will contribute to our understanding of the barriers and facilitators to disability disclosure at work surrounding non-visible conditions uniquely and how these are experienced by disabled employees in educational workplaces.

### Research questions/objectives

The research question is ‘What are the views and experiences of employees relating to non-visible disability disclosure in education workplaces?’. The study objectives are:

- To explore the approaches and rationales of disability disclosure decisions.
- To explore any perceived barriers and enablers of non-visible disability disclosure.
- To explore disabled employees’ experiences during and following disclosure of a non-visible disability.

### Methods

A scoping review was undertaken to map the literature on staff disability disclosure in education workplaces. The review is guided by scoping review aims and methodology as described by Arskey and O’Malley.^20^ Findings will identify any gaps in the literature and support the summary and dissemination of research to policymakers, employers and employees in education settings.

#### Search strategy

Searches were conducted in seven health and education databases including: MEDLINE, ERIC, PsycINFO, APA PsycArticles Full Text, Scopus, Embase and Educational Administration Abstracts. Google Scholar was also searched for any additional articles that may not have been listed in the selected databases. Research terms and strategies were established by the study team and refined with support from a university information specialist. Included articles were published between 2003 and 2023. The search language was limited to English. Further details and searching hits can be found in Appendix 1.

#### Study selection

The studies were selected based on inclusion and exclusion criteria determined a priori. Relevant articles were focused on the disclosure of non-visible disabilities as defined by the UK Parliament,^4^ where disclosure was the focus of the paper. Papers that included both visible and non-visible disabilities were excluded unless they separately reported on non-visible disability disclosure. Qualitative and quantitative studies were included. Study populations were employees aged 18 years or older and working in any department or job role within education employment settings. Education workplaces are defined as nursery/pre-school, children aged 4–18 (primary and secondary), college and further education, higher education, adult education, special educational settings. Non-visible disabilities are defined as a physical, mental or neurological condition(s) that are not visible, or are not immediately observable or apparent, and can limit or pose challenges to an individual’s movements, senses or activities.^4^ Disclosure is defined as ‘formally or informally telling colleagues, human resources, line manager, or organisation’. Although there is no strict delineation between visible/non-visible disabilities and individuals may experience a combination of both, to address our specific aims and objectives we excluded studies that did not have a central focus on non-visible health conditions. Reviews and grey literature were also excluded.

#### Charting the data

A data charting tool was created by following a guideline for scoping reviews developed by Pollock *et al*.^21^ MY established the data charting tool based on the research objectives, and J.H. and H.B. revised it. The tool included the following information: author, publication date and place, title, aim, study design, population, sample size, settings, types of disability, disclosure experiences and disclosure related outcomes.

#### Collating, summarizing and reporting results

Scoping reviews establish a thematic construction from the extant literature in a narrative and descriptive manner.^22^ A narrative review was conducted for knowledge synthesis. This approach enables the opportunity to explore relationships in the data and compare findings using different methodologies. The scoping review objectives guided the analysis of the included papers, focusing on several key aspects related to invisible disability disclosure. These aspects included formal and informal methods of disclosing non-visible disabilities, examining both positive and negative experiences associated with disability disclosure, identifying facilitators and barriers influencing the disclosure process and understanding the reasons behind individuals’ decisions to either disclose or not disclose their disabilities.

## Results

### Study inclusion

The initial search yielded a total of 2531 records from various databases, and an additional 106 records were identified through Google Scholar and reference lists, bringing the combined total to 2637 records. After removing duplicates, 1899 unique records remained for further assessment.

The title and abstract review were conducted on all 1899 records, and 1816 were excluded during this stage with reasons specified. The primary reasons for exclusion were non-relevant content with disability disclosure (1494 records), studies focusing on the student population (162 records) and studies not related to education workplaces (148 records). Additionally, 12 studies were excluded as it focused on individuals with visible disabilities.

Following the title and abstract review, 83 records were selected for full-text review. During this phase, 66 records were excluded based on predetermined criteria. The main reasons for exclusion at this stage were studies not related to education workplaces (28 records), studies focusing on the student population (17 records), studies focusing on the prevalence,^7^ studies not related to disability disclosure.^6^ Furthermore, seven studies one review focused on visible disabilities were excluded.

**Fig. 1:**
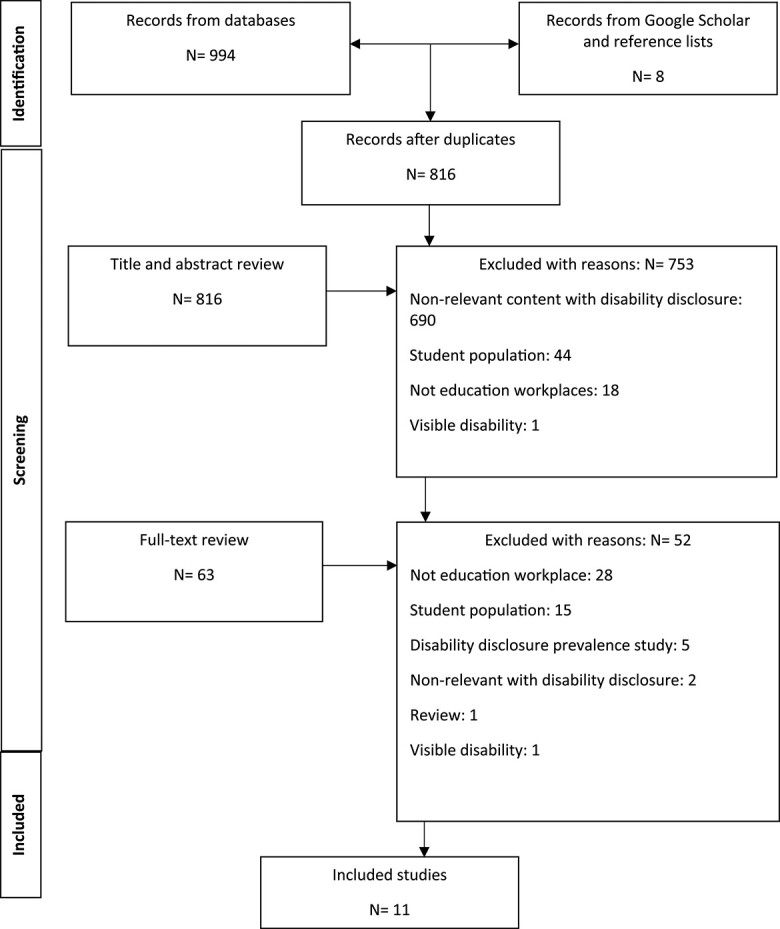
Search results and study selection.

Ultimately, 17 studies met the inclusion criteria and included in the scoping review. Figure 1 represents the flow of screening process.

### Characteristics of included studies

The included studies were conducted across five countries: eight studies were from the USA,^13^^,^^23–29^ four from the UK,^30–33^ three from Canada,^34–36^ one from New Zealand^37^ and one from Germany.^38^ The publication years of the studies ranged from 2009 to 2023. Detailed characteristics of each study can be found in Table 1.

**Table 1:** Characteristics of the included studies

Author Date Country	Title	Aim	Study designs	Population	Sample size	Settings	Disability type	Disclosure type	Disclosure experience	Key outcomes
Price *et al*. 2017 USA^13^	Disclosure of Mental Disability by College and University Faculty: The Negotiation of Accommodations, Supports, and Barriers	To address the lack of research and understanding of the experiences of faculty members with mental disabilities in higher education	Survey	College and university members across USA	267	College or University	Mental disabilities	Formal and informal	Reasons for disclosing: • To request accommodations • To seek support • To reduce stigma and build trust Reasons for not disclosing: • Fear of negative consequences • Stigma • Difficulty finding supportive colleagues or supervisors • Lack of awareness about mental health issues Various positive and negative experiences reported in the disclosing process.	Whilst most faculty with mental disabilities disclosed to colleagues, many lacked awareness of available accommodations and feared negative consequences. Disclosure experiences ranged from positive support to harmful bias, highlighting the need for better institutional support and inclusive workplaces.
Burns and Green 2019 USA^23^	Academic Librarians’ Experiences and Perceptions on Mental Illness Stigma and the Workplace	To understand the stigma and address a gap in the literature about how academic librarians, many of whom are faculty on a tenure track, may experience mental illness stigma in their professional environments.	Survey including free text questions	Academic librarians including 311 diagnosed with a mental illness	549	The survey was distributed amongst American Library Association Listservs.	Mental health problems	Informal	Stigma • expected to ‘work harder’. • seen as suspicious and ‘taking advantage of the system’. • Fear of isolation	Training and workshops can reduce stigma.
Pionke 2019 USA^24^	The Impact of Disbelief: On Being a Library Employee with a Disability	To explores the accommodation process, its impact on the employee and the politics and psychology of disbelief and suspicion surrounding disability accommodation.	Case study	A librarian	1	N/A	Post-traumatic stress disorder (PTSD)	Formal	• Stigma • Long procedure for accommodation Ableism	Accommodation, from whichever angle you approach it, is not an easy thing. Done right, it leads to happier and more dedicated employees who work more efficiently. Done wrong, accommodations create resentment, a sense of betrayal and a devaluing of the self for the person who is asking for them. Whilst the law is clear that accommodations must be offered to people who ask for them, the law does not stipulate that employers have to understand, educate or embrace the person with a disability and that is the crux of the issue.
Cepeda M 2021 USA^25^	Thrice Unseen, Forever on Borrowed Time: Latina Feminist Reflections on Mental Disability and the Neoliberal Academy	To explore the experiences of multiply marginalized faculty members with mental disabilities in the neoliberal academy through a Latina feminist testimonial approach	Autoethnography	Professor	1	Williams College	Bipolar and PTSD	Formal	• Disclose for securing reasonable workplace accommodation and provide support for other colleagues with non-visible disabilities • Stigma • Fear of losing job • Pressure to prove herself and value at work.	The study advocates for a more inclusive and supportive environment for faculty with non-visible disabilities, emphasizing the need for collective recognition and systemic change within academia to accommodate the diverse experiences of academics with mental health conditions and challenges. She urges for a shift in the discourse surrounding disability in higher education and calls for a more holistic approach to support the needs of faculty members with non-visible disabilities.
Green *et al*. 2020 USA^26^	Teaching and Researching with a Mental Health Diagnosis: Practices and Perspectives on Academic Ableism	To examine the experiences of academics with mental health diagnoses in the teaching and research process	Interviews	Academics with mental health diagnoses	9	University settings	Mental health conditions	Formal and informal	Reasons for disclosing: • Creating open dialogue and reducing stigma • Obtaining accommodations • Building trust and empathy Reasons for not disclosing: • Fear of discrimination and stereotyping • Maintaining personal privacy • Varied positive and negative experiences, including challenges in navigating stigma, accessing accommodations and maintaining academic productivity. Positive experiences include support and understanding by colleagues and students, personal empowerment and raising awareness.	Emphasizes the need for a more inclusive and supportive environment for academics with mental health diagnoses in the academy.
England 2016 USA^27^	Being open in academia: A personal narrative of mental illness and disclosure	To present an autobiographical reflection on the decision to be open about the authors’ mental health status during all stages of her career, from diagnosis as a graduate student through the tenure process to her present state of working to attain full professor	Narrative autobiography	A professor in Geography	1	Department of Geography, Miami University.	Bipolar	Formal	Prefer to disclose because of: • A belief needing support from friends and colleagues (safety) • A belief mental illness should be destigmatised. (stigma)	Chronic mental illness is a challenge to disclose in academia. But, universities are becoming more aware of mental health issues and are providing counselling services and programmes to students and staff.
Clayton 2009 USA^28^	Teacher with a Learning Disability	To explore disability experiences of a teacher who discloses a learning disability to her Principal	Case study	A teacher	1	The Northern City Public School	Learning disability	Formal	• Fear of losing job • Low performance is not because of lack of preparation	It is important to disclose the disability. But there are many views on how disable teachers can continue the job.
Valle *et al*. 2010 USA^29^	The Disability Closet: Teachers with Learning Disabilities Evaluate the Risks and Benefits of ‘Coming Out’	To investigates the factors that influence whether teachers with learning disabilities (LD) choose to disclose their disability status within public school settings	Interview	K-12 special education teacher and student teacher	4	N/A	Learning disability	Informal	• Stigma • Fear of losing status as an authority • Some disclosed to only students and their families. (to help others gain a deeper, more positive understanding of LD)	The act of disclosing LD is a not an event, but a highly personal process, subject to a multitude of ongoing factors and always without finalization. The research reveals persistent misperceptions about LD amongst educators, leaving some teachers with LD to feel vulnerable and thus remain undisclosed.
Wood and Happe 2023 UK^30^	What are the views and experiences of autistic teachers? Findings from an online survey in the UK	To discover views and experiences of autistic people working in an education role in the school sector in the UK	Survey (analysis of free text questions)	School staff	149	UK	Autism	N/A	• Fear of losing job • Some lost their job • Ableism (prejudice) • Stigma • Positive and supportive experience	The present findings suggest that autistic staff working in an education role in schools in the UK experience several impediments to their effective and successful employment in the sector. Some participants have positive experiences after disclosing their autism diagnosis, becoming valued members of the school community.
Marshall *et al.* 2020 UK^31^	“What should I say to my employer… if anything?”- My disability disclosure dilemma	To explore the key issues surrounding teacher/staff disability disclosures in the UK’s further education (FE) sector	Semi-structured interviews	Staff	15	Further Education setting in the Southeast of England	Non-visible disabilities including mental conditions	N/A	• Disclosing is anxious, distressing. • Seen as incompetence. • Seen as deficit	Fear of stigma and negative consequences leads most FE teachers to not disclose disabilities. Teachers with disabilities fear discrimination, lack of promotion or job loss if they disclose.
Horton and Tucker 2014 UK^32^	Disabilities in academic workplaces: experiences of human and physical geographers	To explore how diverse disabilities intersect with academic careers, lifestyles and workplaces, focusing on some common disciplinary and institutional spaces of human and physical geography.	Survey with free text questions	Academic staff	75	Respondents from different countries	Mental health conditions	Informal	• Stigma • Competitive working environment • Having clout helps to disclose disabilities • Fear of job lost	There is a need to support those with mental health conditions in academic workplaces. They mostly encountered issues including isolation, lack of support, distress, pressure, low self-esteem, fear of appearing ‘weak’—overlapped with the often-undisclosed experiences of many ‘non-disabled’ colleagues.
Hiscock and Leigh 2020 UK^33^	Exploring perceptions of and supporting dyslexia in teachers in higher education in STEM	To explore the perceptions of dyslexia and the experiences of teachers with dyslexia in higher education in STEM	Mixed methods, online survey and interviews	Teachers in higher education	115	Higher education institutions	Dyslexia	Informal	Disclosing dyslexia to assess students and colleagues’ perceptions. Positives: • Student acceptance • More inclusive and supportive learning environment • Normalizing disability • Encouraging others Negatives: • Stigma • Fear of judgement or negative consequences	Teachers with dyslexia find acceptance from students and colleagues when being open about their diagnosis. Openness and inclusive practices foster trust and understanding, making higher education more equitable for academics with dyslexia.
Skogen 2012 Canada^34^	‘Coming into Presence’ as Mentally Ill in Academia: A New Logic of Emancipation	To discusses the impact of stigma on a professor’s decision to either disclose or conceal her illness.	Autoethnography	Professor	1	University of Alberta	Bipolar	Informal	• Sigma • Fear • Shame	Disclosing a severe mental health issue is a challenging process because of fear, stigma and shame.
Oud 2019 Canada^35^	Systemic Workplace Barriers for Academic Librarians with Disabilities	To explore the workplace experiences of librarians with disabilities working in university libraries in Canada	Interviews	Librarians	10	Canadian university libraries	Non-visible disabilities including mental conditions	Formal and informal	• Disclose as a coping strategy or increasing awareness of disability. • Not disclose because of stigma fear of job or promotion lost. • Mixed positive and negative experiences on legal work accommodations	The main barriers reported in the study were related to a lack of awareness or ill-informed view of disability, including an assumption that everyone in the workplace is nondisabled and negative stereotypes of people with disabilities as lazy and less productive at work.
Morrison 2019 Canada^36^	(Un)Reasonable, (Un)Necessary and (In)Appropriate: Biographic Mediation of Neurodivergence in Academic Accommodations	To critically examine the institutional demands for personal disclosure and the bureaucratic processes involved in securing workplace accommodations for disabled faculty members in higher education	Autoethnography	Associate Professor in English at the University of Waterloo	1	University	ADHD	Informal	• Disclosing to promote a positive shift in the disclosure of disabilities • The extensive and complex nature of the verification process • The fear of being split into an ‘otherwise qualified’	The study explores the challenges faced by disabled faculty in higher education, focusing on the difficulties of disclosure and accommodation. It emphasizes the dehumanizing verification process, bureaucratic emphasis on essential duties and conflicts between institutions and individuals in securing accommodations. The study advocates for a more holistic approach to rebuilding higher education to support access for disabled individuals.
Wright and Kaupins 2018 New Zealand^37^	‘What About Us?’ Exploring What It Means to Be a Management Educator With Asperger’s Syndrome	To explore Asperger’s Syndrome impact on teaching and learning from the instructor’s perspective	Case study - Interview	Professor	1	Boise State University	Asperger’s syndrome (AS)	Prefer not disclose	• No disclosure but fight with that	AS need not be seen as a disability or deficiency in the management classroom. Using cognitive and behavioural techniques, individuals with AS can effectively manage their symptoms, leading to enhanced teaching delivery and assessment methods.
Sanchez 2023 Germany^38^	Decisions, practices and experiences of disclosure by academics with invisible disabilities at German universities	To examine the decisions, practices and experiences of disclosure amongst academics with non-visible disabilities at German universities	Interviews	Academics with non-visible disabilities at German universities	16	University settings	Non- visible disabilities including mental conditions	N/A	Prefer not to disclose because of: • Fear of stigma and discrimination • Concerns about professional competence • Maintaining personal privacy and boundaries	Academics with non-visible disabilities often feel pressured to present themselves as academically capable and competent individuals, selectively sharing and controlling disability information as an anti-stigma strategy within the abled-normative academia. Emphasizes the need for a supportive and inclusive environment for academics with non-visible disabilities in German universities.

The study designs employed in these included studies were diverse, and included five qualitative interviews,^26^^,^^29^^,^^31^^,^^35^^,^^38^ four surveys,^13^^,^^23^^,^^30^^,^^32^ three case studies,^24^^,^^28^^,^^37^ three autoethnography,^25^^,^^34^^,^^36^ an autobiography^27^ and a mixed-method.^33^ Sample sizes varied significantly, ranging from 1 to 549 participants. These studies explored education settings such as universities and colleges,^13^^,^^25–27^^,^^32–34^^,^^36–38^ public schools^28–31^ and academic libraries (i.e. in higher education settings).^23^^,^^24^^,^^35^ The occupational groups examined included university or college members^13^ including professors,^25^^,^^27^^,^^28^^,^^34^^,^^36^^,^^37^ academics,^26^^,^^38^ lecturers,^32^ librarians,^23^^,^^24^^,^^35^ teachers^28^^,^^29^^,^^33^ and school staff.^30^^,^^31^ The studies focused on various non-visible disabilities, including mental health conditions,^13^^,^^23–27^^,^^31^^,^^32^^,^^34^^,^^35^^,^^38^ learning disabilities and differences^28^^,^^29^^,^^33^^,^^36^, Autism^30^ and Asperger’s syndrome.^37^

### Approaches and rationales of non-visible disability disclosure

Across the 17 studies, 12 explored the approaches used by employees in educational workplaces to disclose their disability.^13^^,^^23–29^^,^^32^^,^^33^^,^^35^^,^^36^ Observed across the studies, the employed approaches used for disability disclosure were diverse, included a variety of stakeholders (line managers, co-workers, students and their parents) and did not always include interacting with established human resource (HR) and/or in-house occupational health (OH) systems. We categorized these approaches as either formal^13^^,^^24–28^^,^^35^ or informal^13^^,^^23^^,^^26^^,^^29^^,^^32–36^ forms of disability disclosure at work.

We define a ‘formal’ disclosure approach as one that refers to explicitly informing the employer or institution about one’s disability through official channels or documentation. For employees in educational workplace settings, this process was characterized by following a formal HR procedure^8^ and a formal meeting with management^13^^,^^25^^,^^26^^,^^28^^,^^35^ to discuss workplace accommodations and adaptations. In contrast, we define informal disclosure as sharing information about one’s disability outside of formal HR/OH systems. For employees in educational workplace settings, this disclosure process was characterized by selectively revealing their disabilities to trusted colleagues, students and their parents.^13^^,^^23^^,^^26^^,^^27^^,^^29^^,^^32^^,^^35^^,^^36^

Ten studies explore the rationales for disclosure amongst employees in educational workplace settings.^13^^,^^24–29^^,^^32^^,^^33^^,^^35^ The rationale discussed were multifaceted (influenced by both current and past experiences) and often characterized by instrumental- and/or emotional-directed coping strategies. The main reported reason for formally disclosing a disability to an employer was to access reasonable workplace accommodations.^13^^,^^24–26^^,^^35^ Across both formal and informal forms of disclosure, the other rationales discussed for disability disclosure by employees in education workplaces were the need/want for peer and emotional support^13^^,^^24^^,^^26^^,^^28^^,^^32^ at work, and the desire to raise awareness and promote increased inclusion within and across their work environment.^13^^,^^25^^,^^27^^,^^33–35^ These stated rationales were characterized across formal and informal forms of disclosure. This suggests, perhaps, that in educational workplaces, employees’ disclosure of non-visible disability (within and outside HR systems) is important beyond just accessing reasonable adjustments and securing instrumental needs. It may also yield psychological value through increased opportunities for emotional support, and positive feelings associated with being agents of positive change.

A stated rationale, unique to formal disclosure, was reporting a past positive experience in disclosing their disability in the workplace.^35^ This highlights the importance of considering employees culmination of experiences in the workplace, both past and present, and how this may influence decision-making process and employee behaviours regarding disability disclosure. Potentially unique to employees in educational workplace settings—who chose not to formally declare their disability to their employer—was the nature of the disability itself,^23^^,^^29^ and their perceptions regarding its attached social stigma and anticipated workplace discrimination post-disclosure.

### Employee experiences during and following disability disclosure.

We observed that the lived experience of disabled employees within educational workplace settings, during and following, disability disclosure was complex, and typically characterized by both positive^13^^,^^23^^,^^27^^,^^30^^,^^33^^,^^35^^,^^36^^,^^38^ and/or negative experiences.^13^^,^^23–25^^,^^27^^,^^30^^,^^32^^,^^34–36^^,^^38^ Such experiences were explored in 12^13^^,^^23–25^^,^^27^^,^^30^^,^^32–36^^,^^38^ of the 17 studies. Although findings were mixed, the studies predominantly revealed negative experiences associated with disability disclosure, rather than positive ones.

Among those studies that explored positive experiences^13^^,^^23^^,^^25^^,^^27^^,^^30^^,^^33^^,^^35^^,^^36^^,^^38^ during or following disclosure, they were—typically—characterized by disabled employees feeling as though their instrumental and emotional needs were actively considered and addressed by their workplace. This included employees in educational workplace settings considering that their act of disclosure resulted in workplace accommodations and adaptations that met their expressed needs,^13^^,^^23^^,^^27^^,^^30^^,^^33^ and were implemented in a timely and responsive manner with the necessary resources.^13^^,^^35^ Disabled employees who felt they received support and understanding from their supervisor and colleagues^13^^,^^23^^,^^27^^,^^33^ expressed this as a positive experience. In the study by Wood and Happe,^30^ some (but not all) participants who disclosed their autism at work felt as though they received better understanding and appreciation from the school community and families, leading to a more autism-friendly and accommodating work environment. In England’s^27^ (2002) autobiographical study, a professor reported positive experiences following formal disclosure because of support obtained from colleagues, and the instrumental support from a professional mentor in obtaining requested reasonable adjustments and gaining emotional support. Price *et al*. conducted a survey of college and university staff with a mental health condition across the USA. They found participants reported varied levels of support from their managers and colleagues with a generally positive reception of their disclosure.^13^ A higher number of people reported positive experiences with colleagues and chairs, whereas a lower number reported positive experiences with HRs.^13^ Hiscock and Leigh^33^ found support after dyslexia disclosure encompassed positive colleagues and student feedback including their understanding and perceptions towards teaching with dyslexia. This positive feedback led to an inclusive and supportive working environment. These positive experiences amongst disabled employees in educational workplace settings appear to be shaped by two key considerations. First, the importance of workplace accommodations and reasonable adjustments tailored to the unique needs and expressed wishes of the disabled employee, which are enacted upon by the organization in a purposeful, timely and responsive manner. Second, the importance of also considering what job resources (e.g. mentoring and coaching) and forms of social support (e.g. peer support network, sensitive and informed line managers) can support the disabled employee—during and following—their disability disclosure.

Many of the reviewed studies explored negative experiences^24^^,^^30^^,^^32^^,^^34^^,^^35^ for disabled employees during and beyond disability disclosure. These negative experiences were characterized by challenges in accessing and obtaining requested reasonable adjustments.^24^^,^^35^ In particular, some of the key challenges highlighted included a perceived reluctance of supervisors or management to provide requested workplace accommodations (particularly changes in working patterns and hours^35^), with lengthy waits for adjustments that were not necessarily aligned with what had been agreed.^24^ In Pionke’s^24^ case study the employee felt the wider context of the implemented workplace accommodation (e.g. access to an enclosed office) was not considered. Whilst they were provided with an enclosed office, it was physically located away from her department, resulting in decreased access to social and professional networks and, in turn, increased feelings of social isolation. In this same case, the disabled employee felt disenfranchised and ‘othered’ by management concealing her disability without her consent following her disclosure. A common experience observed across reviewed studies for disabled employees in educational workplace settings was encountering stigma and perceived discrimination following their disclosure from both colleagues^34^ and managers.^30^^,^^32^ Often leading to feelings of invalidation^30^^,^^32^ and ‘othering’,^24^ feeling insecure or replaceable in their professional roles^30^^,^^32^ or being fearful or risk to their career or reputation by disclosing.^34^

### Perceived barriers and enablers of invisible disability disclosure

‘Enablers’ of disability disclosure varied amongst employees with non-visible disabilities. The reasons for disclosure were often influenced by their perceived work environment, support systems and personal goals. Some disabled employees in the reviewed studies chose to disclose to raise awareness about disability issues and to advocate for better conditions for individuals with disabilities in the workplace.^13^^,^^26^^,^^27^^,^^34^^,^^35^ Believing that visibility and openness regarding non-visible disabilities may help to generate a more inclusive and supportive workplace culture. Disabled employees who felt supported, respected and secure in their jobs were more likely to disclose.^13^^,^^23^^,^^26^^,^^30^^,^^32^^,^^35^^,^^36^ A positive and inclusive work environment encouraged employees to feel comfortable sharing information about their disabilities.^13^^,^^23^^,^^30^^,^^32^ Some participants selectively disclosed their disabilities to a few co-workers they trusted and felt safe with.^35^ Selective disclosure allowed them to seek support and assistance without exposing themselves to potential risks that were perceived to be associated with broader or formal forms of disclosure. For some, disclosing their disability was a coping strategy to ensure that colleagues would understand their needs and potential challenges better, reducing misunderstandings or negative judgements.^13^^,^^23^^,^^26^^,^^32^^,^^33^^,^^35^^,^^37^^,^^38^ In several studies, participants felt sharing their diagnosis or health-related experiences with colleagues, students and parents would provide positive role models for others.^30^^,^^34^ They hoped to break stereotypes about non-visible disabilities and show that success and disability are not mutually exclusive. In a few studies,^25^^,^^26^ participants also believed that disclosing their disability helped reduce stigma related to their disability and build trust and empathy with institution,^25^^,^^26^ HRs.^26^ Price’s^13^ study suggested that certain and clear disability disclosure processes may encourage faculty members to share their mental health disabilities with, particularly, HRs and managers. In certain cases, participants chose to disclose their disabilities, particularly their neurodiversity (e.g. autism) and specific learning differences (e.g. dyslexia), only to students and their families rather than their employer.^29^^,^^30^ This disclosure was driven by a desire to promote a deeper, more positive understanding of neurodiversity and specific learning differences, with the intention that this would assist others in similar situations. Job status also impacted on disability disclosure, since those with greater status and seniority felt more secure about their job and, therefore, more confident to disclose a disability.^32^

‘Barriers’ to disability disclosure were prevalent and, broadly, influenced by individuals’ want to keep their disabilities hidden because of fear of stigma, discrimination and ableism. One of the primary barriers to disability disclosure was the fear of negative consequences to career or professional reputation because of anticipated stigma and discrimination.^13^^,^^25^^,^^26^^,^^35^^,^^36^^,^^38^ Across the reviewed studies disabled employees reported being fearful of losing their job or being passed over for promotion^25^^,^^28^^,^^30^^,^^32^^,^^35^ or fear of losing status and authority^29^ as key barriers to disclosing. For example, Horton and Tucker^32^ found that early career academics and researchers expressed insecurity and feelings of replaceability within their departments and institutions.

Fear of isolation in the working environment was also another reason to be reluctant to disclose, which may result from poorly implemented reasonable adjustments^3^ or socially by feeling ‘othered’ through or by this declaration process.^24^^,^^36^ In several studies, the complexity, length and cumbersome nature of access reasonable adjustments and workplace accommodations were a key barrier to disability disclosure.^23^^,^^24^^,^^31^^,^^32^^,^^35^^,^^36^^,^^38^ In one study amongst librarians, many were reluctant—in particular—that gaining access to accommodation requests was contingent on the individual manager, with some reluctant to implement any discussed adjustments.^35^ Previous negative experiences with disability disclosure^29^^,^^31^^,^^35^, competitive working environments,^32^ the fear of being seen to be taking advantage of system^23^ and the fear of being viewed as incompetent^25^^,^^31^^,^^35^^,^^36^^,^^38^ were other reasons for not disclosing disabilities in education workplaces. In a case study,^37^ the participant did not see a pressing need to disclose his disability. They felt that their condition was not debilitating enough to warrant mentioning and preferred to manage their condition privately without seeking workplace accommodations. Maintaining personal privacy and boundaries was reported as reasons for not disclosing.^26^^,^^38^ Several studies^13^^,^^31^^,^^38^ found that employees in education settings found it easier to disclose and discuss a physical disability that was visually apparent, as opposed to disabilities that were not visible to others.

## Discussion and conclusion

The reasons underpinning disclosure are complex and emotive-in-nature. In educational workplace settings, there exists a disability disclosure gap.^16^ As non-visible disabilities can often be concealed by employees, the process of declaring and discussing this individual experience or health condition is highly sensitive^39^ and, in turn, poses unique challenges to organizational leadership.^9^ For example, this impacts on how employing organizations support open discussions surrounding inclusion, which, in turn, impairs opportunities to providing practical support regarding reasonable adjustments tailored to individual wants and needs.^9^ There is a growing trend of non-visible disabilities within the workplace. It is imperative, therefore, to understand the barriers and facilitators to disability disclosure within workplace settings. Particularly, in industries where disability is under-represented.

This scoping review highlights the complex nature of disclosure of a non-visible disability within educational workplace settings. This complex and multifaceted decision-making process is not unique to educational workplace settings but appears to be uniquely experienced across the community of employees with non-visible disabilities.^40–43^ Our review observes both individual and socio-environmental factors appear to influence this decision and process. Ongoing stigma and ableism in the workplace strongly underpin disabled employees’ decision to disclose (or not), to whom, how and when. These are prevalent themes observed across conditions,^41^ as well as across sectors and workplaces^44^.

We conclude that the disability disclosure dilemma—that is the decision to disclose either formally to the organization through HRs systems or management or informally to co-workers—appears to include a personal process of risk evaluation shaped by ableism considerations. This observation is in line with the emerging literature,^40^^,^^43^ which suggests that the decision to disclose includes careful consideration and balancing of perceived risks and costs in comparison to gains and benefits.^45^ When gains and benefits (e.g. increased support and understanding, access to reasonable adjustments) appear to outweigh the potential risks and costs (e.g. feeling undervalued or insecure in their job or position) to the disabled employee, it is likely this will facilitate and enable disclosure (either formally or informally).

**Fig. 2:**
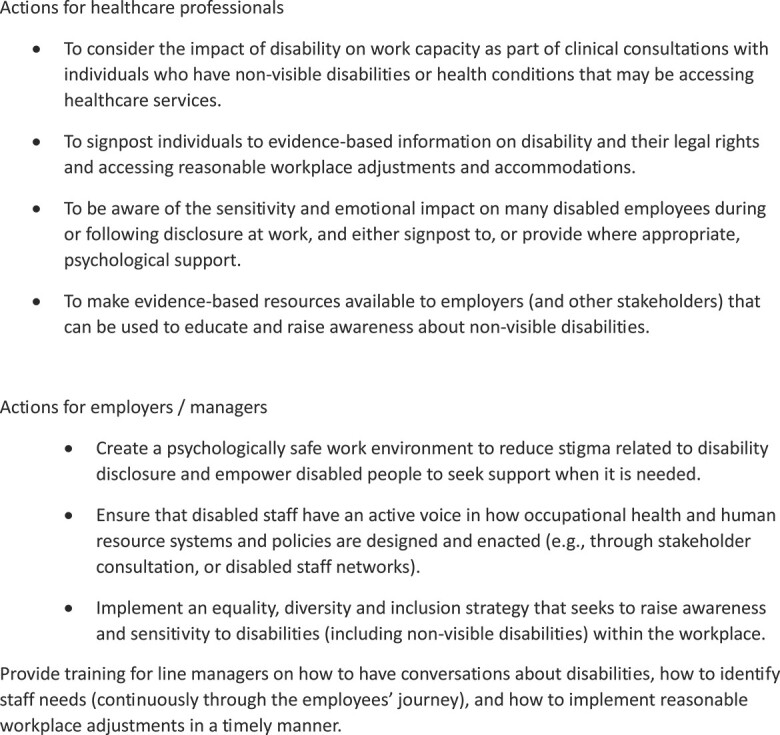
Recommendations for practice.

This process of risk evaluation is dynamic and influenced by both past experiences, but also by the changes in the individual’s role in the organization (e.g. becoming more senior) or health condition (e.g. fluctuations or increased severity), changes in management perceptions and practices (e.g. line manager sensitivity training), evolving working conditions and culture (e.g. flexible work schedules) and availability of support networks (e.g. disabled staff network). Efforts in the education sector to facilitate an inclusive environment for individuals with a non-visible disability have typically focused on students, rather staff.^2^^,^^46^ Therefore, to ensure educational workplaces are inclusive and supportive of disability requires initiatives and supports that target both students and staff collectively and equitably. Both healthcare professionals and employers can play an important role in tackling low levels of disability disclosure in education settings (particularly those with non-visible disabilities) and supporting those who choose to disclose and seek workplace adjustments. Recommendations are outlined in Fig. 2.

## Author contributions

Juliet Hassard (Conceptualization, Investigation, Methodology, Writing—original draft, Writing—review & editing), Mehmet Yildrim (Data curation, Formal analysis, Writing—original draft), Louise Thomson (Conceptualization, Writing—review & editing) and Holly Blake (Conceptualization, Methodology, Project administration, Writing—review & editing)

## Conflict of interest statement

The authors have no potential conflicts of interest.

## Data availability

The authors confirmed that the data supporting the findings of the study are available within the article and its supplementary materials.

## Supplementary Material

Appendix_1_ldae004

## A. Appendix 1

**Searching strategy**

**Table:** OVID including Embase, MEDLINE, PsycINFO, APA PsycArticles Full Text

#	Query	Results from November 8, 2023
1	((invisible or hidden or undisclosed or non-apparent or unseen or concealed or non-evident or mental) adj5 (disability)).mp. [mp = ti, ab, hw, tn, ot, dm, mf, dv, kf, fx, dq, bt, nm, ox, px, rx, an, ui, sy, ux, mx, tc, id, tm, tx, sh, ct]	1206
2	exp disability/	187 750
3	1 or 2	188 841
4	workplace.mp. [mp = ti, ab, hw, tn, ot, dm, mf, dv, kf, fx, dq, bt, nm, ox, px, rx, an, ui, sy, ux, mx, tc, id, tm, tx, sh, ct]	210 266
5	(‘education workplace’ or ‘academic institution’ or ‘university’ or ‘college’ or ‘higher education’ ‘faculty’ or ‘academic setting’ or ‘educational environment’).mp. [mp = ti, ab, hw, tn, ot, dm, mf, dv, kf, fx, dq, bt, nm, ox, px, rx, an, ui, sy, ux, mx, tc, id, tm, tx, sh, ct]	2 351 067
6	4 or 5	2 539 710
7	(‘employee perspectives’ or ‘worker experiences’ or ‘faculty views’ or ‘staff attitudes’ or ‘academic perceptions’ or ‘professional experiences’ or ‘teacher views’).mp. [mp = ti, ab, hw, tn, ot, dm, mf, dv, kf, fx, dq, bt, nm, ox, px, rx, an, ui, sy, ux, mx, tc, id, tm, tx, sh, ct]	7385
8	(‘barriers’ or ‘facilitators’ or ‘experiences’ or ‘views’ or ‘difficulties’ or ‘challenges’ ‘ableism’).mp. [mp = ti, ab, hw, tn, ot, dm, mf, dv, kf, fx, dq, bt, nm, ox, px, rx, an, ui, sy, ux, mx, tc, id, tm, tx, sh, ct]	3 274 558
9	7 or 8	3 277 011
10	3 and 6 and 9	**1633**

**Table:** EBSCOhost including ERIC and Educational Administration Abstracts

#	Query	Results from EBSCOhost on November 8, 2023
1	Disability	166 569
2	disclosure or revealing or reporting or declare or sharing	89 828
3	education or academic or university or college or ‘higher education’ or teacher or lecturer or professor or staff	2 979 838
4	barrier or facilitator or experience or view or difficulty or challenge or accommodation or ableism	869 139
5	invisible or mental or unseen or hidden or undisclosed or concealed or non-apparent or non-evident	162 033
6	1 and 2 and 3 and 4 and 5	**308**

**Table:** Scopus

#	Query	Results from Scopus on November 8, 2023
1	‘Disability disclosure’ OR ‘disability revealing’ OR ‘disability reporting’ OR ‘disability declare’ OR ‘disability sharing’	832
2	(education OR academic OR university OR college OR ‘higher education’ OR teacher OR lecturer OR professor OR staff)	57 577 642
3	(barrier OR facilitator OR experience OR view OR difficulty OR challenge OR accommodation OR ableism)	22 938 932
4	(invisible OR mental OR unseen OR hidden OR undisclosed OR concealed OR non-apparent OR non-evident)	4 620 914
5	1 and 2 and 3 and 4	**590**

**Table:** Google Scholar (screened the first 100 articles), searched through a tool (Publish or Perish 8)

#	Query	Results from Google Scholar on November 8, 2023
1	‘disability disclosure’ OR ‘disclosure experiences’ OR ‘barriers to disability disclosure’ OR ‘facilitators to disability disclosure’ AND ‘invisible disabilities’ OR ‘mental disabilities’ AND ‘education workplaces’ OR ‘higher education’ AND ‘staff’ OR ‘academic staff’	**100**
