# Supplementary material for: Disclosing non-visible disabilities in educational workplaces: a scoping review
Source: Br Med Bull. 2024 Mar 2;150(1):23–41. doi: 10.1093/bmb/ldae004 (PMC11180548; doi:10.1093/bmb/ldae004)
Supplement: Appendix_1_ldae004 [file appendix_1_ldae004.docx]

**Appendix 1**

**Searching Strategy**

OVID including Embase, MEDLINE, PsycINFO, APA PsycArticles Full Text

| # | Query | Results from 8 Nov 2023 |
| --- | --- | --- |
| 1 | ((invisible or hidden or undisclosed or non-apparent or unseen or concealed or non-evident or mental) adj5 (disability)).mp. [mp=ti, ab, hw, tn, ot, dm, mf, dv, kf, fx, dq, bt, nm, ox, px, rx, an, ui, sy, ux, mx, tc, id, tm, tx, sh, ct] | 1206 |
| 2 | exp disability/ | 187750 |
| 3 | 1 or 2 | 188841 |
| 4 | workplace.mp. [mp=ti, ab, hw, tn, ot, dm, mf, dv, kf, fx, dq, bt, nm, ox, px, rx, an, ui, sy, ux, mx, tc, id, tm, tx, sh, ct] | 210266 |
| 5 | ("education workplace" or "academic institution" or "university" or "college" or "higher education" "faculty" or "academic setting" or "educational environment").mp. [mp=ti, ab, hw, tn, ot, dm, mf, dv, kf, fx, dq, bt, nm, ox, px, rx, an, ui, sy, ux, mx, tc, id, tm, tx, sh, ct] | 2351067 |
| 6 | 4 or 5 | 2539710 |
| 7 | ("employee perspectives" or "worker experiences" or "faculty views" or "staff attitudes" or "academic perceptions" or "professional experiences" or "teacher views").mp. [mp=ti, ab, hw, tn, ot, dm, mf, dv, kf, fx, dq, bt, nm, ox, px, rx, an, ui, sy, ux, mx, tc, id, tm, tx, sh, ct] | 7385 |
| 8 | ("barriers" or "facilitators" or "experiences" or "views" or "difficulties" or "challenges" "ableism").mp. [mp=ti, ab, hw, tn, ot, dm, mf, dv, kf, fx, dq, bt, nm, ox, px, rx, an, ui, sy, ux, mx, tc, id, tm, tx, sh, ct] | 3274558 |
| 9 | 7 or 8 | 3277011 |
| 10 | 3 and 6 and 9 | **1633** |

EBSCOhost including ERIC and Educational Administration Abstracts

| # | Query | Results from EBSCOhost on 08 Nov 2023 |
| --- | --- | --- |
| 1 | Disability | 166569 |
| 2 | disclosure or revealing or reporting or declare or sharing | 89828 |
| 3 | education or academic or university or college or "higher education" or teacher or lecturer or professor or staff | 2,979,838 |
| 4 | barrier or facilitator or experience or view or difficulty or challenge or accommodation or ableism | 869,139 |
| 5 | invisible or mental or unseen or hidden or undisclosed or concealed or non-apparent or non-evident | 162,033 |
| 6 | 1 and 2 and 3 and 4 and 5 | **308** |

Scopus

| # | Query | Results from Scopus on 08 Nov 2023 |
| --- | --- | --- |
| 1 | "Disability disclosure” OR “disability revealing" OR "disability reporting" OR "disability declare" OR "disability sharing" | 832 |
| 2 | (education OR academic OR university OR college OR "higher education" OR teacher OR lecturer OR professor OR staff) | 57,577,642 |
| 3 | (barrier OR facilitator OR experience OR view OR difficulty OR challenge OR accommodation OR ableism ) | 22,938,932 |
| 4 | (invisible OR mental OR unseen OR hidden OR undisclosed OR concealed OR non-apparent OR non-evident ) | 4,620,914 |
| 5 | 1 and 2 and 3 and 4 | **590** |

Google Scholar (screened the first 100 articles), searched through a tool (Publish or Perish 8)

| # | Query | Results from Google Scholar on 08 Nov 2023 |
| --- | --- | --- |
| 1 | "disability disclosure" OR "disclosure experiences" OR "barriers to disability disclosure" OR "facilitators to disability disclosure" AND "invisible disabilities" OR "mental disabilities" AND "education workplaces" OR "higher education" AND "staff" OR "academic staff" | **100** |
